# The Effect of Nutrition on Dry Age-Related Macular Degeneration: A Systematic Narrative Review of Evidence and Clinical Implications

**DOI:** 10.7759/cureus.106966

**Published:** 2026-04-13

**Authors:** Ali Istanaksai, Khaled Jama, Tarek Hammadieh, Abdalla Ali, Eesa Siddiqui, Saeed Azizi

**Affiliations:** 1 General Surgery, King's College London, London, GBR; 2 Trauma and Orthopaedics, King's College London, London, GBR; 3 Stroke, Epsom Hospital, Epsom, GBR; 4 Medicine, King's College London, London, GBR; 5 Ophthalmology, Moorfields Eye Hospital NHS Foundation Trust, London, GBR

**Keywords:** age-related macular degeneration, amd, amd treatment, dry age-related macular degeneration, nutrition and diet in age-related macular disease (amd)

## Abstract

Age-related macular degeneration (AMD) is a progressive retinal condition characterised by degeneration of the macula, leading to central vision loss. Whilst the underlying mechanism of AMD is not fully understood, oxidative stress is believed to be the main driving force behind retinal damage. Although the prevalence of AMD increases with factors such as age, smoking, and sun exposure, these alone do not fully explain individual risk. Nutritional factors, in particular dietary antioxidant intake, have been shown to influence the progression of dry AMD by mitigating oxidative stress and supporting retinal cellular function. By considering the effects of nutrition on retinal health and disease outcomes, we can further our understanding of AMD pathogenesis.

## Introduction and background

Age-related macular degeneration (AMD) is the leading cause of irreversible visual impairment in the developed world [1,2]. It predominantly affects individuals over the age of 55 years. The annual incidence of AMD in those aged 55-59 years is 0.3 per 1,000, rising to 36.7 per 1,000 in individuals aged 90 years and above [3]. AMD ranges from mild to severe vision loss and primarily affects high-acuity central vision, which is responsible for tasks such as reading, driving, and recognising faces [4,5]. Beyond age, modifiable factors such as smoking, poor diet, and excessive sun exposure are strongly implicated [6]. The projected global prevalence of 288 million cases by 2040 [1,3] underscores the urgent need for preventive strategies, particularly dietary interventions. There are two categories of AMD, based on the type of damage to the macula. ‘Wet’ or neovascular AMD involves the growth of abnormal new blood vessels under the macula. Although this form is less common, it is associated with a poorer prognosis and accounts for nearly 80% of severe vision loss caused by AMD [1]. It is typically treated using intravitreal vascular endothelial growth factor inhibitors [2]. The more common form is ‘dry’ AMD, which results from degeneration of the retinal pigment epithelium (RPE) due to lipofuscin accumulation [2]. Unlike neovascular AMD, there is no definitive gold standard treatment for dry AMD [2,5]. However, research has shown that the progression of early-stage disease can be slowed with the use of high-dose zinc and antioxidant vitamin supplements [6]. This article aims to review the current state of knowledge on the effect of nutrition on dry AMD, with a specific focus on the components used in the Age-Related Eye Disease Studies (AREDS) formula.

Pathogenesis of dry AMD

The pathogenesis of dry AMD is complex and not yet fully understood. It is believed that oxidative stress, along with genetic, environmental, and inflammatory factors, contributes to damage in the outer layers of the retina, including the RPE, Bruch's membrane, the choriocapillaris, and the choroid [1,2,7]. The products of oxidative stress are subsequently deposited in otherwise healthy tissue, leading to cellular dysfunction. These deposits also impede the exchange of nutrients between blood vessels and the retina, resulting in photoreceptor degradation [2]. Over time, metabolic waste products accumulate in the form of drusen beneath the retina, further impairing photoreceptor nutrition and, consequently, phototransduction [1,2]. Several therapeutic strategies are currently under investigation, attempting to target its underlying pathogenesis [7].

AREDS

The AREDS clinical trials remain among the largest and most significant studies to date, focusing on the effect of antioxidant intake on AMD progression. A total of 3,640 participants were enrolled and divided into four groups based on disease severity. The study concluded that consumption of the AREDS formula reduced the risk of progression by 25% over five years in patients with intermediate AMD or advanced disease in one eye. The original formulation consisted of beta-carotene (15 mg), vitamin C (500 mg), vitamin E (400 IU), zinc (80 mg as zinc oxide), and copper (2 mg, included to prevent copper deficiency associated with high zinc intake). Beta-carotene was later replaced with lutein and zeaxanthin in the updated formula due to the increased risk of lung cancer in smokers [8].

## Review

Methods

**Figure 1:**
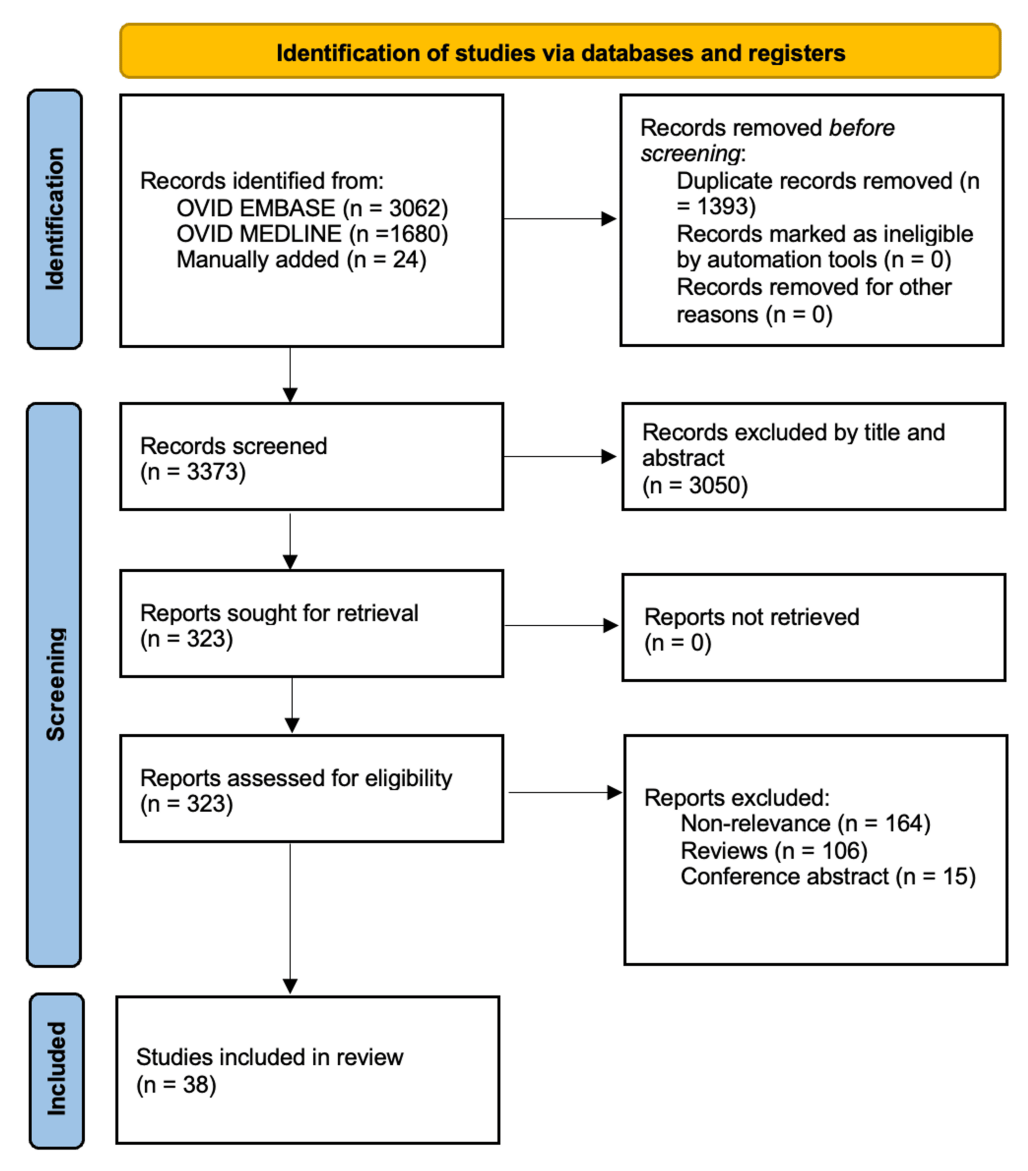
PRISMA flow chart diagram PRISMA, Preferred Reporting Items for Systematic Reviews and Meta-Analyses.

This study followed the guidelines set out by the Preferred Reporting Items for Systematic Reviews and Meta-Analyses (PRISMA) (Figure 1). A systematic search was conducted using the Ovid Embase and Ovid MEDLINE electronic databases in April 2025. The primary aim of this review was to evaluate the effects of dietary factors and nutritional supplementation on the development and progression of dry AMD. The primary search terms were "Age-related macular degeneration" and "diet", supplemented by keywords such as "zinc" and “antioxidants”. Boolean operators "AND" and "OR" were used to combine search terms strategically. Reference lists of relevant articles were also manually reviewed to identify additional eligible studies. References were exported to Mendeley for duplicate removal, resulting in 3,373 articles for screening. Eligibility criteria included publications from 1974 onward, written in English, and encompassing both human and clinically relevant non-human studies. No restrictions were placed on study design or geographical location. Only original research articles were included, while meta-analyses and conference abstracts were excluded. Each selected paper was reviewed independently by a reviewer. Any disagreements between reviewers were resolved through discussion, and where consensus could not be reached, an additional author was consulted. After applying these criteria, a total of 38 studies were selected for inclusion. Risk of bias was assessed using tools appropriate to the study design. Randomised controlled trials (RCTs) were evaluated using the Cochrane risk of bias tool (Table 1). Observational studies were assessed using the Newcastle-Ottawa Scale* *(Table 2)*. *Animal- and laboratory-based studies were assessed qualitatively and discussed among the authors. Tables of our search strategy and PICO framework have been included in the appendix. 

**Table 1:** Risk of bias assessment of randomised controlled trials using the Cochrane risk of bias tool

Serial number	Study	Random sequence generation	Allocation concealment	Blinding of participants/personnel	Blinding of outcome assessment	Incomplete outcome data	Selective reporting	Overall risk
1	The Age-Related Eye Disease Study Research Group, 2013 [8]	Low risk	Low risk	Low risk	Low risk	Low risk	Low risk	Low risk
11	Johnson et al., 2008 [9]	Low risk	Unclear risk	Low risk	Low risk	Low risk	Low risk	Low risk
22	Newsome et al., 1988 [10]	Low risk	Unclear risk	Low risk	Low risk	Unclear risk	Low risk	Moderate risk
23	Newsome et al., 2008 [11]	Low risk	Unclear risk	Low risk	Low risk	Low risk	Low risk	Low risk
24	Stur et al., 1996 [12]	Low risk	Low risk	Low risk	Low risk	Low risk	Low risk	Low risk
37	Christen et al., 2012 [13]	Low risk	Low risk	Low risk	Low risk	Low risk	Low risk	Low risk

**Table 2:** Risk of bias assessment of observational studies using the Newcastle-Ottawa Scale

Serial number	Study	Study type	Selection	Comparability	Outcome/exposure	Total stars
2	Keeling et al., 2022 [14]	Animal model	N/A	N/A	N/A	N/A
3	Barreto et al., 2023 [15]	Observational population study	3	2	3	8
4	Vishwanathan et al., 2016 [16]	Human tissue cross-sectional study	N/A	N/A	N/A	N/A
5	Bone et al., 2001 [17]	Case-control study	3	1	2	6
6	Barker et al., 2011 [18]	Animal model	N/A	N/A	N/A	N/A
7	Seddon et al., 2001 [19]	Prospective cohort study	4	2	3	9
8	Seddon et al., 2006 [20]	Prospective cohort study	4	2	3	9
9	Chiu et al., 2009 [21]	Observational cohort analysis	4	2	3	9
10	Christen et al., 2011 [22]	Prospective cohort study	4	2	3	9
12	Parekh et al., 2009 [23]	Observational cohort study	4	2	3	9
13	Eckhert et al., 1983 [24]	Animal model	N/A	N/A	N/A	N/A
14	Newsome et al., 1996 [25]	Case-control laboratory study	3	1	2	6
15	Leung et al., 2012 [26]	Laboratory experimental study	N/A	N/A	N/A	N/A
16	Tate et al., 1999 [27]	Laboratory cell culture study	N/A	N/A	N/A	N/A
17	Kennedy et al., 1994 [28]	Laboratory cell culture study	N/A	N/A	N/A	N/A
18	Erie et al., 2009 [29]	Case-control laboratory study	3	1	2	6
19	Mares-Perlman et al., 1996 [30]	Observational cohort study	4	2	3	9
20	Tan et al., 2008 [31]	Prospective cohort study	4	2	3	9
21	van Leeuwen et al., 2005 [32]	Prospective cohort study	4	2	3	9
25	Kuzniarz et al., 2002 [33]	Observational cohort study	4	2	3	9
26	Klein et al., 2008 [34]	Observational cohort study	4	2	3	9
27	Nan et al., 2011 [35]	Molecular laboratory study	N/A	N/A	N/A	N/A
28	Stephens et al., 1988 [36]	Animal model	N/A	N/A	N/A	N/A
29	Hayes et al., 1974 [37]	Animal model	N/A	N/A	N/A	N/A
30	Amemiya et al., 1981 [38]	Animal model	N/A	N/A	N/A	N/A
31	Belda et al., 1999 [39]	Observational case-control/cross-sectional study	3	1	2	6
32	Simonelli et al., 2002 [40]	Case-control biomarker study	3	1	2	6
33	Delcourt et al., 1999 [41]	Observational cohort study	4	2	3	9
34	Mares-Perlman et al., 1995 [42]	Population-based case-control study	4	2	2	8
35	Chiu et al., 2009 [43]	Observational cohort analysis	4	2	3	9
36	Age-Related Eye Disease Study Research Group, 2007 [44]	Case-control study	4	2	2	8
38	Shen et al., 2012 [45]	Case-control biomarker study	3	1	2	6

Lutein and zeaxanthin

Both lutein and zeaxanthin are pigments produced by plants and belong to a group of carotenoids called xanthophylls. These pigments are structural isomers of each other and are closely related in terms of function, particularly regarding eye health. They are found in high concentrations in foods such as spinach, broccoli, peas, and parsley. As these pigments are not naturally synthesized in the human body, a daily intake of 10 mg of lutein and 2 mg of zeaxanthin is recommended [46].

Adequate consumption of these carotenoids is considered vital for the normal functioning of bodily organs such as the eyes, heart, and intestines. This is especially important in the context of eye disease, as there is a well-established link between the consumption of unhealthy, high-fat, and cholesterol-rich foods and the development of AMD [14]. While the commonly termed ‘Western diet’ is a risk factor for AMD, a ‘Mediterranean diet’ is considered protective. In fact, a study by Barreto et al. [15] revealed that adhering to a Mediterranean diet reduces the risk of developing AMD by up to 60% in genetically predisposed individuals. In contrast, those who did not adhere to this diet experienced a fourfold increase in AMD risk [15]. The Mediterranean diet consists of limited red meat consumption, replaced by large amounts of fruits, vegetables, whole grains, fish, and healthy fats, all natural sources of vitamins, antioxidants, lutein, and zeaxanthin. The main protective mechanism of these carotenoids against AMD is their ability to scavenge free radicals and reduce the effects of macular oxidative stress. This strong antioxidant ability stems from the conjugated double bonds present in their cyclic ring structure. Other nutrients also play a key role in managing macular oxidative stress, but it has been reported that lutein and zeaxanthin are more effective at managing oxidative stress than agents such as vitamin E. Notably, zeaxanthin has a greater singlet oxygen quenching rate constant than lutein due to the presence of an extra conjugated double bond (11 vs 10).

In the eye, lutein and zeaxanthin are found exclusively in the retina and lens. These pigments are referred to as macular pigments, as they are the only carotenoids to accumulate in the centre of the retina, called the macula. This retinal region contains the highest concentration of lutein and zeaxanthin and consequently demonstrates the greatest resilience to degenerative changes. The central macula, called the fovea, consists mainly of zeaxanthin, while lutein accumulates primarily in the peripheral regions of the macula [16]. Whilst the lens is paramount in absorbing most UV radiation, wavelengths above 400 nm are readily transmitted to the back of the eye where the retina is located. Retinal damage caused by visible light is highly dependent on several factors, including wavelength, energy levels, and exposure time. Blue light (400-500 nm) is particularly harmful to the human eye, as it carries more energy than longer wavelengths. Given their structure, lutein and zeaxanthin can absorb harmful doses of this radiation and thus protect the retina from photooxidative damage. These pigments essentially act as a blue-light filter and prevent oxidative retinal damage by directly quenching free radical species such as singlet oxygen [47].

The protective role of carotenoids in the macula is further illustrated by an inverse correlation between macular pigment density and the incidence of AMD, supporting the importance of dietary carotenoid supplementation in AMD prevention [17]. The AREDS2 randomised clinical trial, completed in 2013 as a continuation of the original AREDS trial (2001), provides essential guidance on dietary supplementation in AMD. Data from AREDS2 revealed that adding lutein and zeaxanthin in a 5:1 ratio to the original AREDS1 formula decreased the progression of AMD to advanced stages by 31% compared with the placebo group. The original AREDS1 formula, which did not contain lutein or zeaxanthin, showed only a 25% reduction. The AREDS2 study also reported increased serum lutein and zeaxanthin concentrations of 190%-210% over the five-year period, while these levels remained unchanged in the placebo group [46].

Omega-3 fatty acids

Omega-3 fatty acids are a type of polyunsaturated fat found in fish and plant-based products such as flaxseeds. The human body is unable to synthesise omega-3 fatty acids, which are of three main types: eicosapentaenoic acid (EPA), docosahexaenoic acid (DHA), and alpha-linolenic acid (ALA). The exact mechanism by which omega-3 may protect against AMD is not fully understood, but it has been suggested that n-3 fatty acids help regulate retinal susceptibility to light-induced damage. In particular, DHA is found at remarkably high concentrations in photoreceptor outer segment membranes, which is thought to relate to its role as a precursor for several important neuroprotective factors, such as neuroprotectin D1. This neuroprotective factor is produced by the RPE in response to oxidative stress. DHA also acts as an anti-inflammatory molecule, further supporting the idea that omega-3 fatty acids may have a beneficial role in AMD [18].

Omega-3 fatty acids were not included in the AREDS formula, and epidemiological studies have reported inconsistent results regarding their protective effect in AMD. Despite this, several studies have reported an association between omega-3 fatty acid consumption and slower AMD progression. The inconsistency in their role is well illustrated by the Eye Disease Case-Control Study, which included 349 cases and 504 controls. Data from this study showed a statistically significant increase in retinal protection in subjects with both a high intake of omega-3 fatty acids and a low intake of linoleic acid (an omega-6 fatty acid). However, this trend became non-significant when omega-6 fatty acid levels were considered [19]. Similar results were observed in the US Twin Study of AMD and the AREDS cross-sectional studies, where statistically significant correlations between high omega-3 consumption and reduced AMD risk were only observed in subjects with low omega-6 fatty acid intake [20].

There is also evidence from both prospective studies and an interventional trial supporting the beneficial effects of omega-3 fatty acids in AMD. A prospective analysis of data from 1,837 AREDS participants found that increased DHA and EPA intake was associated with a decreased rate of AMD progression over 12 years [21]. These results are supported by a prospective analysis of data from 38,022 participants in the Women’s Health Study, which found that women in the highest tertile for DHA intake had a 38% reduced risk of AMD compared with those in the lowest tertile [22]. Furthermore, an interventional trial assessed the correlation between daily consumption of 800 mg DHA in 12 women and macular pigment optical density (MPOD). It was found that after two and four months of DHA supplementation, MPOD was significantly increased from baseline levels. This is an important finding, considering that MPOD serves as a marker for retinal health, suggesting that DHA has a protective role against AMD [9].

In contrast to this, the Carotenoids in Age-Related Eye Disease Study found that high intakes of both omega-6 and omega-3 polyunsaturated fats were associated with a higher prevalence of intermediate AMD when analysing data from 1,787 participants. Specifically, increasing levels of omega-6 were found to double the risk of intermediate AMD. A similar linear trend was observed between high omega-3 intake and the risk of developing intermediate AMD, although it was noted that the positive effects of omega-3 consumption may have been masked by high omega-6 intake [23]. This lack of clarity surrounding the interaction between polyunsaturated fats, combined with conflicting results from different studies, ultimately led to the exclusion of omega-3 fatty acids from the AREDS2 formula [46].

Zinc

Zinc is a vital trace element, and its deficiency has been implicated in a range of physiological impairments and pathological conditions. In terms of the eye, zinc is found in various ocular structures, with particularly high concentrations in the RPE and the retina [24]. This has prompted the idea that zinc may play a crucial role in maintaining retinal health.

In the RPE, zinc is believed to interact with taurine and vitamin A, regulate the light-rhodopsin reaction, and act as an antioxidant [48]. Decreasing levels of endogenous zinc in the RPE are observed with increasing age, as validated by in vitro studies, and the overall concentration of ocular zinc is reduced in eyes showing signs of AMD [25,26]. Ocular signs of AMD in eyes with lower levels of zinc are thought to be due to increased oxidative stress and deficits in metabolic pathways, including phagocytic and lysosomal functions, as well as apoptosis [27,28]. For this reason, it has been suggested that supplementation with zinc may be an effective method for treating or preventing dry AMD.

A cross-sectional analysis of 44 participants indicated a significantly reduced concentrations of copper and zinc in the RPE of individuals with AMD compared with healthy controls [29]. A retrospective analysis of 1,968 participants from the Beaver Dam Eye Study revealed that individuals with the highest dietary zinc intake had a lower risk of developing early AMD than those with the lowest intake [30]. A 10-year prospective analysis of 1,709 patients from the same study showed that those with the highest zinc intake were at significantly reduced risk of developing any type of AMD [31]. These findings are supported by a prospective analysis of 4,170 participants from the Rotterdam Study, which found that increased zinc consumption was associated with a decreased risk for any stage of AMD [32]. Furthermore, an RCT of 90 participants found that supplementation with 100 mg of zinc twice daily for 24 months resulted in significantly increased visual acuity compared with baseline [10]. In another study involving 80 subjects with dry AMD, supplementation with 25 mg of zinc monocysteine twice daily for six months resulted in improved markers of retinal function, including visual acuity and contrast sensitivity [11].

Despite these studies suggesting that zinc supplementation may play a key role in retinal health and AMD, other research has failed to establish a link. This is illustrated by a placebo-controlled trial involving 112 AMD subjects, which found no significant change in vision following two years of daily supplementation with 200 mg of zinc [12]. A large cross-sectional study from the Blue Mountains Eye Study involving 2,873 participants also concluded that zinc had no effect on early AMD [33]. In fact, analysis of the Beaver Dam Eye Study found an increased risk of late AMD associated with zinc supplementation [34]. As mentioned earlier, the RPE is known to have a remarkably high concentration of zinc, likely due to its role in processing photoreceptor outer segments. While studies have shown reduced concentrations of zinc in the RPE of patients with AMD, it has also been suggested that excessively high zinc concentrations may damage the RPE and that genetic factors may influence whether zinc plays a protective or harmful role [35]. Overall, existing results on the benefits of zinc supplementation in AMD are conflicting and inconclusive. This highlights the need for further research into the role of zinc in AMD pathogenesis.

Vitamin E

Vitamin E is a fat-soluble vitamin with antioxidant properties that exists in eight forms, with γ-tocopherol being the most common form in Western plant-based diets. Evidence suggests that oxidative stress may be one of the contributing factors driving neurodegenerative diseases, including retinopathy. As such, it has been proposed that vitamin E may help prevent retinal degeneration. The retina, given its high metabolic rate and rich blood supply, is especially susceptible to oxidative damage from free radical products. Antioxidants play an important role in maintaining the health of retinal tissue and, consequently, overall retinal function. Vitamin E is found in high concentrations within the RPE and the outer portions of photoreceptor cells, where it protects against oxidative injury and retinal oedema [36]. Current guidelines from the Food and Nutrition Board of the Institute of Medicine recommend a daily allowance of 15 mg of vitamin E. A deficiency in vitamin E has been associated with irreversible damage to retinal structure and function, as well as other neurological symptoms. This is well illustrated by a study in which monkeys developed macular degeneration after being placed on a vitamin E-deficient diet [37]. Large focal lesions in the outer photoreceptor segments were also reported in a study investigating vitamin E deficiency in rats [38]. Case-control studies in human populations have found significantly reduced plasma vitamin E concentrations in patients with AMD compared with healthy controls [39,40]. A cross-sectional analysis from the Pathologies Oculaires Liées à l'Âge (POLA) study found an inverse relationship between plasma vitamin E levels and the risk of early AMD development [41]. However, despite substantial evidence supporting the role of vitamin E in AMD, there is also an abundance of conflicting data. For instance, a case-control study involving 167 patients with cataract from the Beaver Dam Eye Study found no relationship between serum concentrations of alpha- and gamma-tocopherol and any stage of AMD [42]. In fact, in the 10-year follow-up of the Blue Mountains Eye Study, participants with the highest vitamin E intake were found to have a greater likelihood of developing late-stage atrophic AMD [31]. Overall, the large volume of conflicting evidence regarding the effect of vitamin E on AMD makes it difficult to draw definitive conclusions about its role in disease prevention or progression.

Vitamin C 

Vitamin C, also known as ascorbic acid, is an antioxidant found in a variety of fruits and vegetables. Despite being included in the AREDS2 formula, the evidence supporting the singular role of vitamin C in AMD is lacking. Two cross-sectional studies of baseline AREDS data suggest that vitamin C has no effect on drusen formation or other types of AMD [43, 44]. This is supported by findings from the Beaver Dam Eye Study, which concluded that prior consumption of vitamin C, either through a normal diet or supplementation, has no significant effect on the risk of early or late AMD [30]. An RCT involving 14,236 male subjects also found no association between AMD risk and daily supplementation with 500 mg of vitamin C over a period of eight years [13]. Although the majority of data support this, a case-control study involving 56 AMD subjects found significantly lower plasma vitamin C levels in patients with AMD compared with healthy controls [45]. However, this was later attributed to the small sample size, and the consensus still stands that the relationship between vitamin C and AMD is weak

Limitations

The searches for this review were limited to two databases, Ovid Medline and Ovid Embase, which may have resulted in the exclusion of relevant studies indexed elsewhere. Furthermore, while some nutrients have been studied extensively, there remains a limited understanding of the role of other specific nutrients in the progression of dry AMD. This is compounded by the presence of conflicting data that limits the strength of the evidence base.

## Conclusions

Various nutritional factors influence the progression of dry AMD. These factors, including lutein, zeaxanthin, omega-3 fatty acids, zinc, and vitamins E and C, have different effects on oxidative stress and retinal health, aiding our understanding of the disease’s pathogenesis. Although consistent evidence supports the protective role of lutein and zeaxanthin, the data for zinc, vitamin E, and omega-3 fatty acids remain inconclusive, often limited by heterogeneity and confounding dietary factors. Importantly, no nutrient alone prevents AMD progression; rather, benefits appear greatest when considered within dietary patterns such as the Mediterranean diet. Future research should prioritise well-powered, genotype-stratified RCTs and longitudinal cohort studies to clarify which subgroups benefit most from supplementation. Translating this evidence into clinical guidelines is critical to optimise prevention and slow AMD progression.

## Appendices

**Table 3:** Search strategy

Database	Date of search	Search terms	Filters	Results
OVID Embase	April 2025	(age-related macular degeneration OR AMD) AND (diet*.ti,ab. OR nutrition.ti,ab. OR zinc/ OR antioxidants/)	Language: English; publication years: 1974-2025	3062
OVID MEDLINE	April 2025	(age-related macular degeneration OR AMD) AND (diet*.ti,ab. OR nutrition.ti,ab. OR zinc/ OR Antioxidants/)	Language: English; publication years: 1974-2025	1680

**Table 4:** PICO table

PICO element	Description
P (population)	Adults with age-related macular degeneration, individuals at risk of developing AMD, and relevant experimental animal or cellular models used to investigate AMD mechanisms
I (intervention/exposure)	Dietary intake, nutritional status, or supplementation of nutrients, including antioxidants, carotenoids, zinc, omega-3 fatty acids, and vitamins associated with AMD
C (comparison)	Placebo, standard diet, lower nutrient intake, absence of supplementation
O (Outcome)	Development or progression of AMD, retinal structural changes, visual function outcomes, or biochemical markers related to oxidative stress, antioxidant status, or retinal physiology

## References

[1] Thomas CJ, Mirza RG, Gill MK (2021). Age-related macular degeneration. Med Clin North Am.

[2] Marchesi N, Capierri M, Pascale A, Barbieri A (2024). Different therapeutic approaches for dry and wet AMD. Int J Mol Sci.

[3] Fleckenstein M, Schmitz-Valckenberg S, Chakravarthy U (2024). Age-related macular degeneration: a review. JAMA.

[4] Guymer RH, Campbell TG (2023). Age-related macular degeneration. The Lancet.

[5] Flores R, Carneiro Â, Vieira M, Tenreiro S, Seabra MC (2021). Age-related macular degeneration: pathophysiology, management, and future perspectives. Ophthalmologica.

[6] Mitchell P, Liew G, Gopinath B, Wong TY (2018). Age-related macular degeneration. The Lancet.

[7] Cabral de Guimaraes TA, Daich Varela M, Georgiou M, Michaelides M (2022). Treatments for dry age-related macular degeneration: therapeutic avenues, clinical trials and future directions. Br J Ophthalmol.

[8] The Age-Related Eye Disease Study Research Group (1999). The Age-Related Eye Disease Study (AREDS): design implications AREDS report no. 1. Control Clin Trials.

[9] Johnson EJ, Chung H-Y, Caldarella SM, Snodderly DM (2008). The influence of supplemental lutein and docosahexaenoic acid on serum, lipoproteins, and macular pigmentation. Am J Clin Nutr.

[10] Newsome DA, Swartz M, Leone NC, Elston RC, Miller E (1988). Oral zinc in macular degeneration. Arch Ophthalmol.

[11] Newsome DA (2008). A randomized, prospective, placebo-controlled clinical trial of a novel zinc-monocysteine compound in age-related macular degeneration. Curr Eye Res.

[12] Stur M, Tittl M, Reitner A, Meisinger V (1996). Oral zinc and the second eye in age-related macular degeneration. Invest Ophthalmol Vis Sci.

[13] Christen WG, Glynn RJ, Sesso HD (2012). Vitamins E and C and medical record-confirmed age-related macular degeneration in a randomized trial of male physicians. Ophthalmology.

[14] Keeling E, Lynn SA, Koh YM (2022). A high fat "Western-style" diet induces AMD-like features in wildtype mice. Mol Nutr Food Res.

[15] Barreto P, Farinha C, Coimbra R (2023). Interaction between genetics and the adherence to the Mediterranean diet: the risk for age-related macular degeneration. Coimbra Eye Study Report 8. Eye Vis (Lond).

[16] Vishwanathan R, Schalch W, Johnson EJ (2016). Macular pigment carotenoids in the retina and occipital cortex are related in humans. Nutr Neurosci.

[17] Bone RA, Landrum JT, Mayne ST, Gomez CM, Tibor SE, Twaroska EE (2001). Macular pigment in donor eyes with and without AMD: a case-control study. Invest Ophthalmol Vis Sci.

[18] Barker FM, Snodderly DM, Johnson EJ, Schalch W, Koepcke W, Gerss J, Neuringer M (2011). Nutritional manipulation of primate retinas, V: effects of lutein, zeaxanthin, and n-3 fatty acids on retinal sensitivity to blue-light-induced damage. Invest Ophthalmol Vis Sci.

[19] Seddon JM, Rosner B, Sperduto RD, Yannuzzi L, Haller JA, Blair NP, Willett W (2001). Dietary fat and risk for advanced age-related macular degeneration. Arch Ophthalmol.

[20] Seddon JM, George S, Rosner B (2006). Cigarette smoking, fish consumption, omega-3 fatty acid intake, and associations with age-related macular degeneration: the US twin study of age-related macular degeneration. Arch Ophthalmol.

[21] Chiu CJ, Klein R, Milton RC, Gensler G, Taylor A (2009). Does eating particular diets alter the risk of age-related macular degeneration in users of the Age-Related Eye Disease Study supplements?. Br J Ophthalmol.

[22] Christen WG, Schaumberg DA, Glynn RJ, Buring JE (2011). Dietary ω-3 fatty acid and fish intake and incident age-related macular degeneration in women. Arch Ophthalmol.

[23] Parekh N, Voland RP, Moeller SM (2009). Association between dietary fat intake and age-related macular degeneration in the Carotenoids in Age-Related Eye Disease Study (CAREDS): an ancillary study of the Women's Health Initiative. Arch Ophthalmol.

[24] Eckhert CD (1983). Elemental concentrations in ocular tissues of various species. Exp Eye Res.

[25] Newsome DA, Miceli MV, Tate DJ, Alcock NW, Oliver PD (1996). Zinc content of human retinal pigment epithelium decreases with age and macular degeneration, but superoxide dismutase activity increases. J Trace Elem Exp Med.

[26] Leung KW, Gvritishvili A, Liu Y, Tombran-Tink J (2012). ZIP2 and ZIP4 mediate age-related zinc fluxes across the retinal pigment epithelium. J Mol Neurosci.

[27] Tate DJ, Miceli MV, Newsome DA (1999). Zinc protects against oxidative damage in cultured human retinal pigment epithelial cells. Free Radic Biol Med.

[28] Kennedy CJ, Rakoczy PE, Robertson TA, Papadimitriou JM, Constable IJ (1994). Kinetic studies on phagocytosis and lysosomal digestion of rod outer segments by human retinal pigment epithelial cells in vitro. Exp Cell Res.

[29] Erie JC, Good JA, Butz JA, Pulido JS (2009). Reduced zinc and copper in the retinal pigment epithelium and choroid in age-related macular degeneration. Am J Ophthalmol.

[30] Mares-Perlman JA, Klein R, Klein BE, Greger JL, Brady WE, Palta M, Ritter LL (1996). Association of zinc and antioxidant nutrients with age-related maculopathy. Arch Ophthalmol.

[31] Tan JS, Wang JJ, Flood V, Rochtchina E, Smith W, Mitchell P (2008). Dietary antioxidants and the long-term incidence of age-related macular degeneration: the Blue Mountains Eye Study. Ophthalmology.

[32] van Leeuwen R, Boekhoorn S, Vingerling JR, Witteman JC, Klaver CC, Hofman A, de Jong PT (2005). Dietary intake of antioxidants and risk of age-related macular degeneration. JAMA.

[33] Kuzniarz M, Mitchell P, Flood VM, Wang JJ (2002). Use of vitamin and zinc supplements and age-related maculopathy: the Blue Mountains Eye Study. Ophthalmic Epidemiol.

[34] Klein BE, Knudtson MD, Lee KE (2008). Supplements and age-related eye conditions: the Beaver Dam Eye Study. Ophthalmology.

[35] Nan R, Farabella I, Schumacher FF (2011). Zinc binding to the Tyr402 and His402 allotypes of complement factor H: possible implications for age-related macular degeneration. J Mol Biol.

[36] Stephens RJ, Negi DS, Short SM, Van Kuijk FJ, Dratz EA, Thomas DW (1988). Vitamin E distribution in ocular tissues following long-term dietary depletion and supplementation as determined by microdissection and gas chromatography-mass spectrometry. Exp Eye Res.

[37] Hayes KC (1974). Retinal degeneration in monkeys induced by deficiencies of vitamin E or A. Invest Ophthalmol Vis Sci.

[38] Amemiya T (1981). Photoreceptor outer segment and retinal pigment epithelium in vitamin E deficient rats. An electron microscopic and electron histochemical study. Albrecht Von Graefes Arch Klin Exp Ophthalmol.

[39] Belda JI, Romá J, Vilela C (1999). Serum vitamin E levels negatively correlate with severity of age-related macular degeneration. Mech Ageing Dev.

[40] Simonelli F, Zarrilli F, Mazzeo S (2002). Serum oxidative and antioxidant parameters in a group of Italian patients with age-related maculopathy. Clinica Chimica Acta.

[41] Delcourt C, Cristol JP, Tessier F, Léger CL, Descomps B, Papoz L (1999). Age-related macular degeneration and antioxidant status in the POLA study. Arch Ophthalmol.

[42] Mares-Perlman JA, Brady WE, Klein R, Klein BE, Bowen P, Stacewicz-Sapuntzakis M, Palta M (1995). Serum antioxidants and age-related macular degeneration in a population-based case-control study. Arch Ophthalmol.

[43] Chiu CJ, Milton RC, Klein R, Gensler G, Taylor A (2009). Dietary compound score and risk of age-related macular degeneration in the age-related eye disease study. Ophthalmology.

[44] Age-Related Eye Disease Study Research Group (2007). The relationship of dietary carotenoid and vitamin A, E, and C intake with age-related macular degeneration in a case-control study: AREDS Report No. 22. Arch Ophthalmol.

[45] Shen XL, Jia JH, Zhao P, Fan R, Pan XY, Yang HM, Liu L (2012). Changes in blood oxidative and antioxidant parameters in a group of Chinese patients with age-related macular degeneration. J Nutr Health Aging.

[46] The Age-Related Eye Disease Study 2 (AREDS2) Research Group (2013). Lutein + zeaxanthin and omega-3 fatty acids for age-related macular degeneration: the Age-Related Eye Disease Study 2 (AREDS2) randomized clinical trial. JAMA.

[47] Narimatsu T, Negishi K, Miyake S (2015). Blue light-induced inflammatory marker expression in the retinal pigment epithelium-choroid of mice and the protective effect of a yellow intraocular lens material in vivo. Exp Eye Res.

[48] Grahn BH, Paterson PG, Gottschall-Pass KT, Zhang Z (2001). Zinc and the eye. J Am Coll Nutr.

